# Supplementary material for: The enterococcal cytolysin synthetase has an unanticipated lipid kinase fold
Source: eLife. 2015 Jul 30;4:e07607. doi: 10.7554/eLife.07607 (PMC4550811; doi:10.7554/eLife.07607)
Supplement: Figure 5—source data 1. — All calculated masses are [M + H]. -: not observed. DOI: http://dx.doi.org/10.7554/eLife.07607.016 [file elife07607s001.docx]

**Figure 5 – source data 1. Calculated and observed masses of CylL_S_ peptides modified by CylM and CylM mutants *in E. coli*.** All calculated masses are [M+H]. - : not observed.

|  | M–4 H_2_O | M–3 H_2_O | M–2 H_2_O | M–H_2_O | M | M–2 H_2_O + HPO_3_ | M–H_2_O + HPO_3_ | M + HPO_3_ | M–2 H_2_O + 2 HPO_3_ | M–H_2_O + 2 HPO_3_ | M+ 2 HPO_3_ | M+ 3 HPO_3_ |
| --- | --- | --- | --- | --- | --- | --- | --- | --- | --- | --- | --- | --- |
| enzyme treatment |  |  |  |  |  |  |  |  |  |  |  |  |
| Calc. | 8331 | 8349 | 8367 | 8385 | 8403 | 8447 | 8465 | 8483 | 8527 | 8545 | 8563 | 8643 |
| CylM | 8331 | - | - | - | - | - | - | - | - | - | - | - |
| CylM-D347A | - | - | - | - | 8401 | - | - | - | - | - | - | - |
| CylM-H349A | 8332 | 8350 | - | 8386 | 8402 | - | - | - | - | - | - | - |
| CylM-N352A | - | - | - | - | 8402 | - | - | - | - | - | - | - |
| CylM-D364A | - | - | - | - | 8403 | - | - | - | - | - | - | - |
| CylM-D252A | - | - | 8364 | 8381 | - | 8444 | 8462 | 8479 | 8525 | 8542 | 8559 | 8640 |
| CylM-H254A | - | - | - | 8384 | 8402 | - | 8464 | 8482 | - | - | - | - |
| CylM-R506A | - | - | - | - | 8402 | - | - | 8482 | - | - | 8562 | - |
| CylM-T512A | - | - | - | - | 8402 | - | - | 8482 | - | - | - | - |
| CylM-K274A | 8332 | - | - | 8385 | 8402 | - | - | - | - | - | - | - |
