# Supplementary material for: The enterococcal cytolysin synthetase has an unanticipated lipid kinase fold
Source: eLife. 2015 Jul 30;4:e07607. doi: 10.7554/eLife.07607 (PMC4550811; doi:10.7554/eLife.07607)
Supplement: Figure 5—source data 3. — All calculated masses are [M + H]. -: not observed. DOI: http://dx.doi.org/10.7554/eLife.07607.018 [file elife07607s003.docx]

**Figure 5 – source data 3. Calculated and observed masses of CylL_S_ peptides incubated with CylM and CylM mutants *in vitro* for 10 h.** All calculated masses are [M+H]. -: not observed.

|  | M–4H_2_O | M–3H_2_O | M–2H_2_O | M–H_2_O | M | M–2H_2_O + HPO_3_ | M–H_2_O + HPO_3_ | M + HPO_3_ | M–2H_2_O + 2HPO_3_ | M–H_2_O + 2HPO_3_ | M+ 2HPO_3_ | M+ 3HPO_3_ |
| --- | --- | --- | --- | --- | --- | --- | --- | --- | --- | --- | --- | --- |
| treatment |  |  |  |  |  |  |  |  |  |  |  |  |
| Calc. | 7061 | 7079 | 7097 | 7115 | 7133 | 7177 | 7195 | 7213 | 7257 | 7275 | 7293 | 7373 |
| CylM | 7061 | - | - | - | - | - | - | - | - | - | - | - |
| CylM-D347A | - | - | - | - | 7133 | - | - | - | - | - | - | - |
| CylM-H349A | 7061 | 7078 | - | - | - | - | - | - | - | - | - | - |
| CylM-N352A | - | - | - | 7114 | 7131 | - | 7195 | - | - | - | - | - |
| CylM-D364A | - | - | - | - | 7133 | - | - | - | - | - | - | - |
| CylM-D252A | - | - | 7096 | 7114 | 7132 | 7176 | 7194 | - | 7257 | 7274 | - | - |
| CylM-H254A | 7061 | - | - | - | - | - | - | - | - | - | - | - |
| CylM-R506A | - | - | - | 7113 | 7133 | - | 7193 | - | - | 7274 | - | - |
| CylM-T512A | - | - | - | - | 7133 | - | - | 7211 | - | - | 7292 | 7372 |
